# Supplementary material for: Conditional chemoconnectomics (cCCTomics) as a strategy for efficient and conditional targeting of chemical transmission
Source: eLife. 2024 Apr 30;12:RP91927. doi: 10.7554/eLife.91927 (PMC11060718; doi:10.7554/eLife.91927)
Supplement: Figure 3—source data 3. [file elife-91927-fig3-data3.zip › Figure 3 source data3 part 1.zip!/Figure 3-Source data 3-part1/1071-1-CRISPResso_Report_osfbjvga.zip!/CRISPResso_on_1071_4/4b.Insertion_deletion_substitution_locations.pdf]

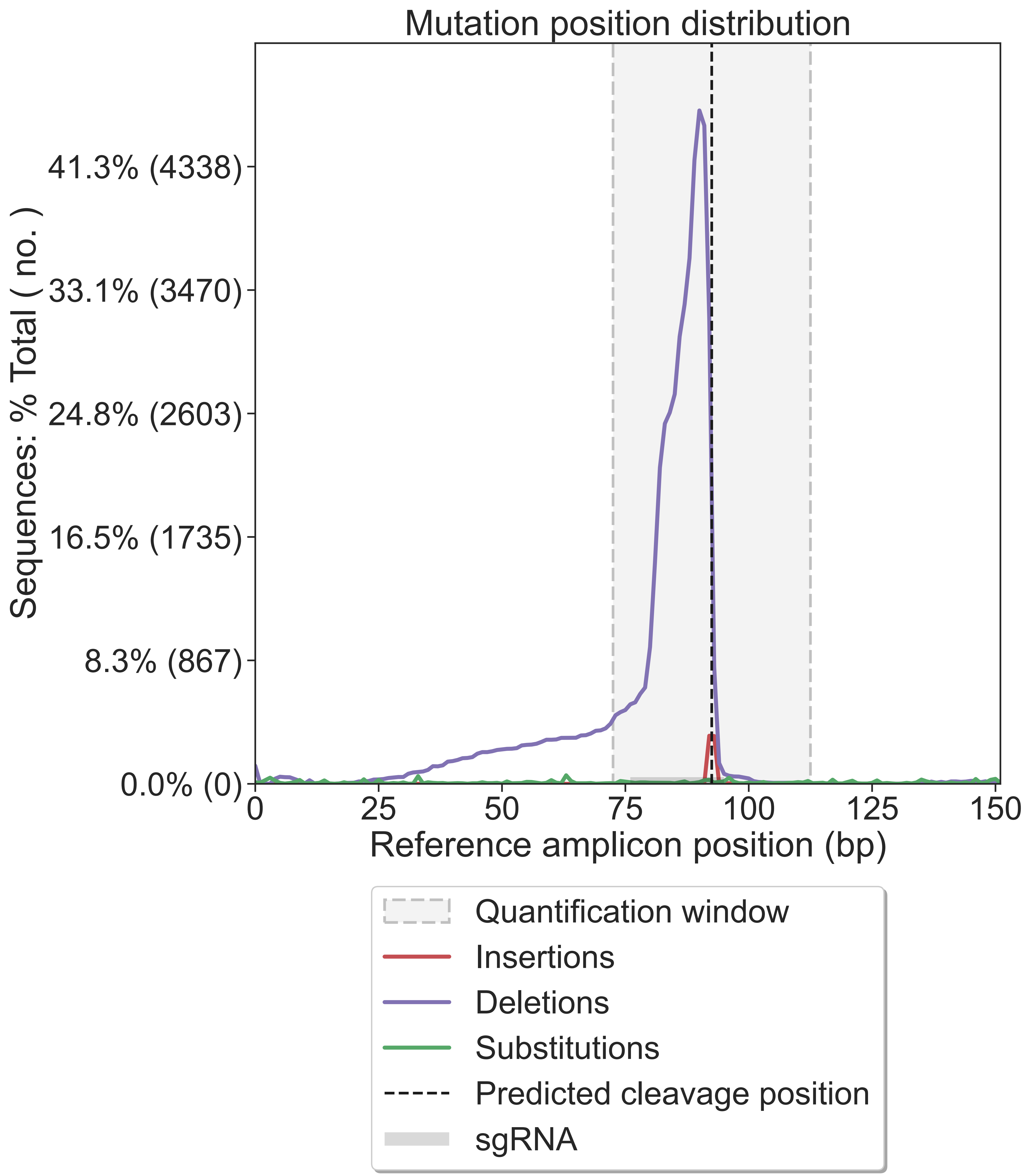

Mutation position distribution
Sequences: % Total (no.)
41.3% (4338)
33.1% (3470)
24.8% (2603)
16.5% (1735)
8.3% (867)
0.0% (0)
0
25
50
75
100
125
150
Reference amplicon position (bp)
Quantification window
Insertions
Deletions
Substitutions
Predicted cleavage position
sgRNA
